# Comparative Genome Analysis of *Bifidobacterium longum* subsp. *infantis* Strains Reveals Variation in Human Milk Oligosaccharide Utilization Genes among Commercial Probiotics

**DOI:** 10.3390/nu12113247

**Published:** 2020-10-23

**Authors:** Rebbeca M. Duar, Giorgio Casaburi, Ryan D. Mitchell, Lindsey N.C. Scofield, Camila A. Ortega Ramirez, Daniela Barile, Bethany M. Henrick, Steven A. Frese

**Affiliations:** 1 Evolve BioSystems, Inc., Davis, CA 95618, USA; rduar@evolvebiosystems.com (R.M.D.); gcasaburi@evolvebiosystems.com (G.C.); rmitchell@evolvebiosystems.com (R.D.M.); lindsey3552@gmail.com (L.N.C.S.); ortega8@hawaii.edu (C.A.O.R.); bhenrick@evolvebiosystems.com (B.M.H.); 2 Foods for Health Institute, University of California at Davis, Davis, CA 95616, USA; dbarile@ucdavis.edu; 3 Department of Food Science and Technology, University of California at Davis, Davis, CA 95616, USA; 4 Department of Food Science and Technology, University of Nebraska, Lincoln, NE 68588, USA

**Keywords:** gut microbiome, dysbiosis, probiotics, HMO, NEC

## Abstract

Dysbiosis is associated with acute and long-term consequences for neonates. Probiotics can be effective in limiting the growth of bacteria associated with dysbiosis and promoting the healthy development of the infant microbiome. Given its adaptation to the infant gut, and promising data from animal and *in vitro* models, *Bifidobacterium longum* subsp. *infantis* is an attractive candidate for use in infant probiotics. However, strain-level differences in the ability of commercialized strains to utilize human milk oligosaccharides (HMOs) may have implications in the performance of strains in the infant gut. In this study, we characterized twelve *B. infantis* probiotic strains and identified two main variants in one of the HMO utilization gene clusters. Some strains possessed the full repertoire of HMO utilization genes (H5-positive strains), while H5-negative strains lack an ABC-type transporter known to bind core HMO structures. H5-positive strains achieved significantly superior growth on lacto-*N*-tetraose and lacto-*N*-neotetraose. *In vitro*, H5-positive strains had a significant fitness advantage over H5-negative strains, which was also observed *in vivo* in breastfed infants. This work provides evidence of the functional implications of genetic differences among *B. infantis* strains and highlights that genotype and HMO utilization phenotype should be considered when selecting a strain for probiotic use in infants.

## 1. Introduction

Hospitalization, surgical birth, antibiotic exposure, and many routine perinatal interventions are known to have detrimental effects on the gut microbiome, resulting in dysbiosis [1]. If unresolved, the negative effects of dysbiosis can be acute and/or long lasting [2,3]. Dysbiosis is linked to the pathogenesis of common neonatal conditions including necrotizing enterocolitis (NEC) and sepsis [4] as well as the development of autoimmune diseases later in life [2,3]. Human milk has been linked to improved infant outcomes with respect to acute and long-term disease risk. These benefits are in part provided by favoring the growth of specific beneficial bifidobacteria capable of consuming human milk oligosaccharides (HMOs) [5].

HMOs are indigestible by the infant, yet they are the third most abundant component in human milk, reaching nearly 20 g/L in colostrum and between 5 and 15 g/L in mature milk [6,7]. Structurally, all HMOs follow common blueprints, which consist of a lactose moiety decorated with either fucose or sialic acid or elongated by a β-1-3/6-linked lacto-*N*-biose (LNT) or lacto-*N*-tetrose (LNnT) to derive type-1 and type-2 core structures, respectively. These core structures can be further elongated with residues of galactose, N-acetylglucosamine, and decorated with fucose or sialic acid [8]. These type-1 and type-2 cores are found in HMO structures representing ~70% of the total HMO concentration in women that express the fucosyl transferase FUT2, known as “secretors” (FUT2^+/+^ or FUT2^+/−^) and over 90% of the total HMO content in “non-secretors” (FUT2^−/−^) [7]. Notably, type-1 HMOs consistently predominate over type-2 HMOs (LNnT core), a trait that appears to be characteristic of human milk [7,9,10,11].

Infant-adapted bifidobacteria have evolved different strategies to assimilate HMOs. *Bifidobacterium breve* and *Bifidobacterium bifidum* deploy the extracellular enzymes and engage in inter- and intra-species tropic networks to sequentially degrade HMOs. [12,13]. In contrast, *Bifidobacterium longum* subsp. *infantis* (*B. infantis*) metabolizes HMOs intracellularly. Accordingly, the genome of *B. infantis* encodes an assortment of genes dedicated to the transport and the intracellular metabolism of HMOs, organized in five gene clusters (H1 to H5) [14], including a contiguous 40 kb cluster (H1) that appears to be conserved amongst all *B. infantis* genomes examined so far [15,16]. Studies using the type strain ATCC 15697 revealed that HMOs are selectively bound through specific extracellular solute binding proteins (F1SBP) and imported into the cell using ABC-type transporters [12,13]. Once internalized, HMOs are hydrolyzed into their monosaccharide components and metabolized via the “bifid shunt” pathway, producing mainly lactate and acetate as fermentation end products [17,18,19]. This transporter-dependent intracellular consumption strategy enables *B. infantis* to efficiently capture preferred carbon sources in the competitive ecosystem of the gut and lowers the intestinal pH through the production of organic acids, which can inhibit the growth of competitors [13,20,21,22,23].

Genotypic variation within HMO utilization gene clusters have been documented across *B*. *longum* subspecies [15]. However, the functional and ecological implications of strain-level differences among commercialized strains have not been explored in detail. The objective of this study was to assess genomic variation among various *B. infantis* isolates, obtained from commercially available probiotic products, by performing a comparative analysis of their genomes. We then determined how the presence or absence of key genes involved in HMO utilization affected the growth of these strains *in vitro*. Finally, in a proof-of-concept experiment, we examined the stool samples of breastfed infants who were fed an H5-positive *B. infantis* probiotic strain (replete with the full gene repertoire of HMO-utilization genes) and an H5-negative *B. infantis* probiotic strain (deplete of an ABC-type transporter in the H5-cluster), in order to measure the relative abundance of each strain recovered in the stool samples.

## 2. Materials and Methods

### 2.1. Bacterial Strains and Culture Conditions

Bacterial strains used in this study (Table 1) were isolated from commercial probiotic products or obtained from the American Type Culture Collection (ATCC.org). Briefly, probiotic products were homogenized, diluted and cultured in *Bifidobacterium* selective iodoacetate mupirocin (BSIM) media prepared as described by Lewis et al. (2016) [24]. Plates were incubated at 37 °C in an anaerobic chamber maintained with a gas mix of 5% H_2_, 5% CO_2_, and 90% N_2_ (Coy Laboratory Products, Grass Lake, MI, USA) for 48–72 h until distinguishable colonies were formed. Resulting colonies were streaked onto BSIM agar and, after two passages, were grown in MRS broth supplemented with 0.05% L-cysteine HCl. The identity of 10 randomly selected colonies per product was determined using Sanger sequencing of the species-variable Internal Transcribed Spacer region within the rRNA locus, housed between the 16S and 23S rRNA genes, using the forward 5′-CTKTTGGGYYCCCKGRYYG-3′ and reverse 5′-CGCGTCCACTMTCCAGTTCTC-3′ primers [25]. For products with multiple strains of *Bifidobacterium*, a selective HMO-modified MRS (hmMRS) agar medium was devised to contain (per liter) 2 g of ammonium citrate, 10 g of tryptone, 2 g of dipotassium phosphate, 0.2 g of magnesium sulfate, 0.05 g of manganese sulfate and 0.5 g of L-cysteine HCL. The 2′-FL solutions (100 g/L) were prepared in distilled water and sterilized by filtration (0.2 µM) and added to autoclaved mMRS to a final 2′-FL concentration of 20 g/L. Strains isolated through this method were confirmed as *B. infantis* using subspecies-specific qPCR as described by Lawley et al. (2017) [26]. Confirmed *B. infantis* strains were stored at −80 °C in MRS containing 15% glycerol. Inocula for growth experiments were prepared by growing individual strains overnight in MRS broth, followed by subculture (with 1% inoculum) in fresh mMRS + 0.5% L-cysteine HCL for 16 h.

### 2.2. Whole-Genome Sequencing Assembly and Annotation

High-molecular-weight (>30 Kb) DNA was extracted from *B. infantis* strains using the MasterPure Gram-Positive DNA Purification Kit (Epicentre, Madison, WI, USA) following the manufacturer’s instructions with an additional lysis step including achromopeptidase (5 U/µL) in conjunction with lysozyme. Extracted DNA was quantified using the Quant-iT™ dsDNA High-Sensitivity Kit (Invitrogen) and checked for integrity in a 1% agarose gel.

Genome sequences were generated by a combination of paired-end Illumina reads and MinION or PacBio long reads. Multiplexed short-read libraries were prepared for all strains excluding BB-02 (DSM 33361) using the Nextera XT Library preparation kit (Illumina, San Diego, CA, USA) and sequences were determined using a 2 × 300 paired-end Illumina MiSeq run at the University of California Davis DNA Technologies Core Facility. A library was prepared for the BB-02 (DSM 33361) strain with NEBNext^®^ Ultra™ DNA Library Prep Kit for Illumina (New England BioLabs, Ipswich, MA, USA) and sequenced with a 2 × 300 paired-end run on an Illumina MiSeq (Illumina, San Diego, CA, USA) by GENEWIZ (South Plainfield, NJ). To generate Oxford Nanopore long reads, the Rapid Barcoding kit (SQK-RBK004; Oxford Nanopore, Oxford UK) was used to prepare barcoded libraries according to the manufacturer’s instructions. The sequencing library was loaded into the flow cell (R9.4.1). A 6 h sequencing protocol was selected on the MinKNOW control software. PacBio sequencing was performed at the Vincent J. Coates Genomics Sequencing Laboratory at the University of California, Berkeley using a Pacific Biosciences RSII sequencer (Pacific Biosciences, Menlo Park, CA, USA). Bacterial genome assemblies were carried out using a hybrid assembly approach combining PacBio or MinION long reads with Illumina reads in Spades v3.11 [27] with parameters optimized for hybrid assemblies [28]. Assembled genomes were annotated with Prokka v1.12 [29] using default parameters.

### 2.3. Genome-Wide Comparisons, Phylogenetic Analysis and HMO Gene Analysis

Bacterial genomes were visualized and compared using the BLAST Ring Image Generator (BRIG) v0.95 [30]. Gene map comparisons were generated with Easyfig [31]. Average nucleotide identity was calculated using the ANIm algorithm from pyANI v.0.2.10 [32]. The annotated genomes obtained with Prokka were then processed with Roary version 3.11.2, [33] to identify the set of core genes (95% protein clustering identity threshold). Unique genes identified by Roary were then manually curated to confirm the uniqueness of genes by strain. For strains predicted to have more than 10 unique genes, a BlastX analysis (cut off: E value of 1 × 10^−4^) was used to confirm presence of homologues in the type strain as previously described [34].

Phylogenetic analysis for assembled genomes was performed within the Anvi’o multi-omics platform v6.2 [35]. Protein sequences of 71 curated single-copy genes [35,36] were used for downstream phylogenetic analysis. Homologs were aligned and concatenated with MUSCLE v3.8.1551 [37]. A phylogenetic tree was generated with FastTree v.2.1.10 [38]. The tree was rooted with an outgroup of *B. longum* subsp. *longum* JCM 1217. HMO utilization genes protein sequences were queried against each genome using local tBLASTn within local Blast v.2.9.0+ [39]. The phylogenetic tree with HMO utilization gene heat map was generated in R v3.6.1 using the phylo.heatmap function within the phytools v0.7-20 package.

### 2.4. Growth on HMO Standards

Probiotic strains were tested for their ability to grow in lacto-*N*-tetraose (LNT), lacto-*N*-neotetraose (LNnT), and 2′-Fucosyllactose (2′-FL) (Glycome A/S, Hørsholm, Denmark) as the sole carbon source. Briefly, on a modified MRS (mMRS) media to contain (per liter) 2 g of ammonium citrate, 10 g of tryptone, 2 g of dipotassium phosphate, 0.2 g of magnesium sulfate, 0.05 g of manganese sulfate and 0.5 g of L-cysteine HCL. Medium components were dissolved in distilled water to 80% of the final volume and autoclaved. Individual HMO standards LNT, LNnT or 2′-FL were dissolved in the residual volume (20%), sterilized by filtration, and added to the autoclaved medium to final concentration of 20 g/L. Media were inoculated at 1% (v/v) using standardized 16 h cultures of each strain. Growth profiles were monitored over 30 h by measuring optical density (OD_600_) using an Epoch2 spectrophotometer (Biotek, Winooski, VT, USA) at 37 °C, placed inside an anaerobic chamber maintained with a gas mix of 5% H_2_, 5% CO_2_, and 90% N_2_ (Coy Laboratory Products, Grass Lake, MI, USA). Three biological replicates were performed for each strain. Growth curve parameters were calculated using the Growthcurver R package v0.2.1 [40].

### 2.5. Purification of HMOs from Human Milk and Bacterial Growth

Purified HMOs were obtained from 50 L of human milk pooled from 11 donors kindly provided by Medolac Laboratories (Boulder City, NV, USA) to Dr. Daniela Barile. An integrated approach for the purification of oligosaccharides, based on optimized conditions that favor elimination of lipids, proteins and simple sugars, was applied with minor modification to adapt the protocol from bovine milk to human milk composition [41]. Maximum lactose hydrolysis and selective fermentation of the resulting monosaccharide was achieved, while oligosaccharides were obtained by the sequential use of pilot-scale membrane filtrations such as ultrafiltration, microfiltration and nanofiltration followed by lyophilization, allowed to obtained an HMO-rich powder that was free of lipids, proteins and simple sugars, with an HMO recovery of nearly 90%. Mono- and disaccharides and the most abundant HMOs ((6′-sialyllactose (6′-SL); 3′-sialyllactose (3′-SL); lacto-*N*-tetraose (LNT); lacto-*N*-neotetraose (LNnT), 3-fucosyllactose (3′-FL), 2′-fucosyllactose (2′-FL), and lacto-N-fucopentaose I (LNFP I)) were quantified by using High-Performance Anion-Exchange Chromatography with Pulsed Amperometric Detection (HPAEC-PAD, ICS-5000, Thermo Scientific, Sunnyvale, CA, USA) following previously published procedures [42]. 

For growth experiments, purified HMOs were dissolved to a concentration of 10 g/L in RPMI 1640 (sans glucose) medium (Corning, Corning, NY, USA). Cultures and growth experiments were conducted as described above.

### 2.6. Glycoprofiling

Bacterial cultures in mMRS medium were collected at 6 and 30 h of growth, representing the early-log phase and the late stationary phase. Bacterial cells were removed by centrifugation at 10,000× *g* for 2 min. Supernatant was collected and diluted 10,000-fold. Dilutions were filtered through a 0.22 μm cellulose acetate membrane (VWR International, Radnor, PA, USA) and 25 µL of these supernatants were injected into a High-Performance Anion-Exchange Chromatograph Coupled with Pulsed Amperometric Detection instrument (HPAE-PAD ICS-5000, Thermo Scientific, Sunnyvale, CA, USA) according to methods from [43] with some modifications. Briefly, chromatographic separation was carried out on a CarboPac PA1 analytical column (4 × 250 mm, DionexTM, ThermoFisher Scientific, Waltham, MA, USA) and CarboPac PA1 guard column (4 × 50 mm, Dionex) with an isocratic gradient, 0–30 min 73.5% A, 25% B, 1.5% C, at a 1.0 mL/min flow rate, where solvent A was deionized water, solvent B 100 mM NaOH and solvent C was 500 mM NaOAc in 100 mM NaOH. HMOs were quantified using calibration curves generated using reference standards of LNT, LNnT (Dextra, Reading, UK) and 2′-FL (Carbosynth, St. Gallen Switzerland), ranging in concentration from 0.00025 to 0.005 mg/mL. All samples were analyzed in triplicate.

### 2.7. Strain-Specific Primer Development

A real-time PCR assay was developed to distinguish and quantify the abundance of the H5-positive strain EVC001 and the H5-negative strains NLS, HA-116 and PI_008. Briefly, a reciprocal BLASTn search was used to compare *B. infantis* EVC001 and H5-negative strains to identify candidate genes containing strain-specific SNPs. The LNB phosphorylase gene (“Blon_2174” in strain ATCC 15697) was identified as a candidate region. Primer3 [44] was used to design the forward 2174_F 5′-GATCGGTGTTGTTGATCACG-3′ and reverse 2147_R 5′-CCTCCCACAACGAAGACAAG-3′ primers as well as the strain-specific probes G1_probe 56-FAM/CAATACAGGCCCTGCTCG-3IABkFQ and G2_probe 56-JOEN/CAGTACAGGCCCTGTGCG-3IABkFQ. To validate the primers, *in silico* PCRs were carried out for all multiplex assays with CLC Main Workbench v.9.5.4 (CLC bio, Aarhus, Denmark) using the ‘Find Binding Sites and Create Fragments’ tool. Specificity of the primers/probe combination was also determined by BLASTn searches against the NCBI nr database and validated experimentally by combining DNA from H5-positive and H5-negative strains. No unspecific amplification was detected. Primer/probe efficiency was calculated for each primer/probe set using five 1:10 serial dilutions of the genomic DNA extracted from a culture for which cell numbers had been determined by plate count.

### 2.8. In Vitro Competitive Assays

Competitive growth of NLS strain and EVC001 was determined by serial subculture over a 72 h period. Three parallel cultures of each strain were initiated by transferring single colonies into 10 mL MRS medium (Difco BD, Franklin Lakes, NJ, USA). To initiate experiments, 16 h cultures of each strain were standardized to an OD_600_ of 1.0. Strains were co-inoculated as a 100-fold dilution in 10 mL of MRS media containing 20 g/L of either LNT, LNnT, 2′-FL or a combination of all three in equal proportions (mixed HMO(mHMO)). Serial passages were performed every 24 h by diluting the culture 1:100, so that 0.1 mL of the medium with bacteria was transferred into a 10 mL of fresh medium containing the same HMO. At each transfer, the cultures were first mixed by vortex for 30 seconds. All incubations were performed at 37 °C inside anaerobic chamber maintained at 5% H_2_, 5% CO_2_, and 90% N_2_ (Coy Laboratory Products, Grass Lake, MI, USA). Cells from 1.0 mL samples were collected from the inoculum and at the end of each incubation period (24, 48 and 72 h) and frozen at −80 °C.

To determine whether the initial inoculum influenced the observed relative fitness of H5-positive strains and H5-negative strains, pairwise growth competition experiments were conducted in 96-well plates. Briefly, 16 h liquid cultures of the competing strains were adjusted to an OD_600_ of 1.0 and washed twice with RPMI medium (sans glucose). Adjusted cultures were then mixed at ratios of 1:1, 1:2 and 1:4 by volume (H5 positive to H5 negative). Mixtures were then used to inoculate 200 μL of RPMI media containing 20 g/L of LNnT or 2′-FL as the sole carbon source. Plates were incubated anaerobically at 37 °C. The competition mixtures were maintained by 100-fold dilutions performed every 24 h, for three cycles. After each cycle, cells from each well were spun down and pellets frozen at −80 °C. 

Genomic DNA was extracted from the cell pellets using a KingFisher flex purification system (ThermoFisher Scientific, Waltham, MA, USA), with reagents from the ZymoBIOMICS 96 MagBead DNA kit (Zymo Research, Irvine, CA, USA). Standard curves were used for absolute quantification of each strain using genomic DNA extracted from previously quantified cultures. Quantitative PCRs were performed on a QuanStudio3 (ThermoFisher Scientific, Waltham, MA, USA). The reaction mixture consisted of 0.5 μL of each primer (10 μM each), 5 μL of PerfeCTa Multiplex qPCR ToughMix (QuantaBio, Beverly, MA, USA), 11.5 μL of water and 5 μL of template DNA. The PCR conditions included 1 cycle of initial denaturation at 95 °C for 3 min, followed by 40 cycles at 95 °C for 15 s and 60 °C for 1 min.

### 2.9. In Vivo Strain Competition Experiments

The relative fitness of the strains EVC001 and NLS in the ecological conditions of the breastfed infant gut was determined over a period of 3 days in a proof-of-concept study. Two (one 5-day-old male and one 3-day-old female) vaginally born, exclusively breastfed infants were fed half a serving of the probiotic product containing the strain *B. infantis* EVC001 and a full serving of the probiotic product containing the strain *B. infantis* NLS. Servings were determined as per the label instructions. Fecal samples were collected one day prior and for three days after the infants were fed the strain combination. DNA extraction and strain quantification were performed as described for the *in vitro* tests. Total *B. infantis* was quantified using methods by Lawley et al. 2017 [26]. Competitive fitness was defined as the proportion of each strain to the total *B. infantis* population calculated as the sum of abundances of both strains in the stool samples. The protocol utilized in this case study (EV-8701) was IRB approved. Informed consent was obtained from all study participants. The original protocol was written to feed equivalent amounts of each strain, but due to commercial availability as well as concentration differences in the powdered probiotics, mothers ended up feeding the servings as described above.

### 2.10. Statistical Analysis

Statistical tests were performed as described in figure legends using R v.3.6.2 or the software GraphPad Prism v.7.0. Differences between groups were considered statistically significant when *p* values were < 0.05.

### 2.11. Data Availability

The novel genome sequences of *B. infantis* strains sequenced in this study were deposited in NCBI (https://www.ncbi.nlm.nih.gov/) under BioProject accession number PRJNA636403. Individual strains accession numbers are listed in Table 1.

## 3. Results

### 3.1. General Genome Features of B. infantis Strains

In order to determine the genomic characteristics of distinct *B. infantis* strains, we sequenced and closed the genomes of 12 isolates obtained from probiotic products targeted for pediatric populations (Table 1). We performed a genome-wide comparative analysis including published *B. infantis* genomes. When strain designation was not noted on the product label, probiotic isolates were assigned integer numbers based on isolation order preceded by a PI_ for Probiotic Isolate. Same strains isolated from different products are denoted by subscripts (x and y) (Table 1). To ensure uniformity in gene prediction, all *B. infantis* genomes, including those retrieved from public databases, were annotated using Prokka v1.12 [29]. The average genome coverage was 202× (min = 88×; max = 486×) (Table 1). The number of predicted coding sequences ranged from 2227 for the *B. infantis* PI_002 to 2547 for the *B. infantis* ATCC 15697 strains, respectively. The percentage G+C content was calculated to be an average of 59.38 ± 0.24%. The average genome size was 2659 ± 97.9 Kbp, with *B. infantis* EVC001 having the largest genome (2832 Kbp) and NLS having the smallest (2598 Kbp). The genomes of ATCC 15697, JCM 1222 and EVC001 were predicted to encode 84 tRNAs while the rest of the strains were predicted to encode 59 (Table 1).

### 3.2. Comparative Genomic Analyses Revealed a High Degree of Genetic Conservation

**Figure 1:**
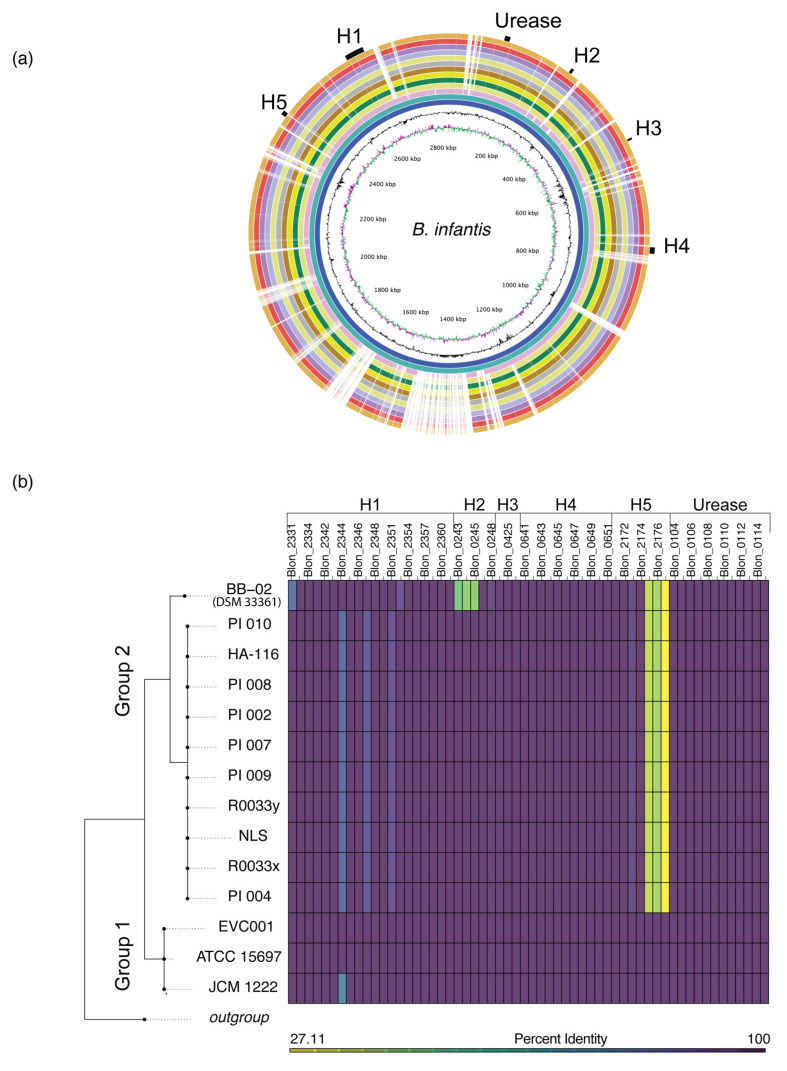
Genomic comparison, phylogenetic relatedness, and human milk oligosacharide (HMO) gene distribution of *B. infantis* strains compared to the type strain, *B. infantis* ATCC 15797. (**a**) Circular map of 13 *B. infantis* strains in comparison to the type strain *B. infantis* ATCC 15697. The innermost rings represent the% G + C (black) and the GC skew (green/purple). HMO-utilization clusters, numbered H1 to H5 and the urease cluster are represented by black boxes next to the outermost ring. The remaining circles display BLASTn searches against the genome of *B. infantis* ATCC 15697. Genomes circles are ordered sequentially based on decreasing average nucleotide identity (ANI) scores (Table S1) beginning with the genome of JCM 1222 (most inner blue circle) and followed by EVC001, BB-02 (DSM 33361), PI_004, PI_007, PI_009, PI_010, NLS, R0033x, PI_002, HA-116, R0033y, PI_008. Homology regions are colored from lightest to darkest shade, respectively. Regions with less than 70% identity appear as blank spaces in each ring. (**b**) Heat map displaying MLST phylogenetic relatedness and percent sequence identity (determined by tBLASTn) of HMO utilization genes compared to the genome of *B. infantis* ATCC 15697. The color of each tile of the heat map indicates the percent homology as indicated by the key. Tiles representing the genes of each cluster are arranged sequentially but, for space constrains, only every other Blon_ locus tag is shown.

The BLAST Ring Image Generator (BRIG) was used to graphically compare strains using the type strain *B. infantis* ATCC 15697 as reference. As shown in Figure 1a, the chromosomal backbones of the strains *B. infantis* EVC001 and JCM 1222 were congruent to the type strain. The remaining strains had large regions of similarity (99%) to the type strain, interspersed with regions of dissimilarity and gaps. Rings were ordered based on ANI scores against *B. infantis* ATCC 15697 in decreasing order from inner (highest score) to outer (lowest score) rings, respectively (max > 0.99; min = 0.98; median = 0.98; ±SD = 0.005). The intensity of color in the figure reflects the percent similarity identity based on BLASTn results against *B. infantis* ATCC 15697, from darker colors representing 100% similarity to lighter colors representing a 70% minimum identity score. GC content and GC sk (+/−) are also reported (Figure 1a).

Examination of the BRIG image and manual curation of genomes aligned using Progressive Mauve [45] revealed gaps and regions of dissimilarity in the sequence alignments which were mainly annotated as hypothetical proteins or mobile genetic elements, including bacteriophage-related proteins and transposases. Particularly, the region between 1600 and 2100 kb contained three major gaps that were classified as incomplete prophage using the PHAge Search Tool Enhanced Release (PHASTER), 2016 release [46]. The main area of gap between the 1370 and 1500 kb region was not classified as prophage by PHASTER and thus manually curated and annotated. A total of 131 open reading frames were identified, of which only 18 were functionally annotated and included transposases, transcriptional regulators, phage integrases and pseudogenes (Figure 1a).

Average nucleotide identity (ANI) was computed for all genomes and resulted in high percent identity overall, with an average ANI of 99.274% (±SD 0.76%). Compared to the type strain, the average ANI for all genomes was 98.79 (±SD 0.68%). Of the probiotic isolates, the genome closest to the ATCC 1567 was EVC001 with an ANI of 99.9%; and the most divergent was PI_008, with an ANI of 98.43% (Table S1).

To identify shared and unique genes, we performed a pangenome analysis using Roary. This analysis highlighted a high level of genome sequence similarity with a few putatively strain-specific genes. The core pangenome consisted of 1621 open reading frames (ORFs). Notably, not all HMO utilization genes were conserved, with notable divergence at the H5 gene cluster in all probiotic isolates except for EVC001. The strain BB-02 (DSM 33361) was predicted to have 481 unique ORFs, of which the majority were annotated as hypothetical proteins (80%). Of the remaining putatively unique genes with a known functional annotation, the BLASTx analysis revealed only 19 genes with no homology to the genome of the type strain. Of those, six were plasmid/phage related and another six had orthologues in the ATCC 15697 or other probiotic *B. infantis* genomes. The remaining eight putatively unique genes to BB-02 (DSM 33361) are spread along the genome and are listed in Table S2. The strain with the second most predicted unique genes was EVC001, having 10 unique ORFs annotated as hypothetical proteins. The remaining strains were highly homogeneous but the strains PI_010, PI_009 and PI_006 were predicted to have three, two, and one unique ORF, respectively. One ORFs was predicted to be unique to the strain R0033x but not found in R0033y, which is intriguing and suggests a sequencing and/or assembly error given that the strains are the same but isolated from different products. Here, it is worth mentioning that slightly different variations in the results on the reported ORFs are expected every time a pangenome is computed, even when starting with the same input. This is due to the fact that Roary uses a non-deterministic heuristic approach to reduce computational time (Page et al., 2015). The remaining strains were not predicted to have any unique ORFs. 

### 3.3. Phylogenetic Grouping Corresponds with Divergence in HMO Utilization Genes

Multi-locus sequence analysis was conducted to gain further insight into phylogenetic relatedness of the strains. Visualization of the phylogenomic tree revealed a clear separation of the strains into two distinct groups, with considerable genetic homogeneity within each group. Group 1 consisted of *B. infantis* EVC001, and the type strain genomes, ATCC 15967 and JCM1222. The remaining isolates formed a monophyletic clade (Group 2) with a remarkable degree of homogeneity between the genomes (Figure 1b). These results corroborate the high degree of genome conservation, as suggested by the pangenome and ANI analyses.

**Figure 2:**
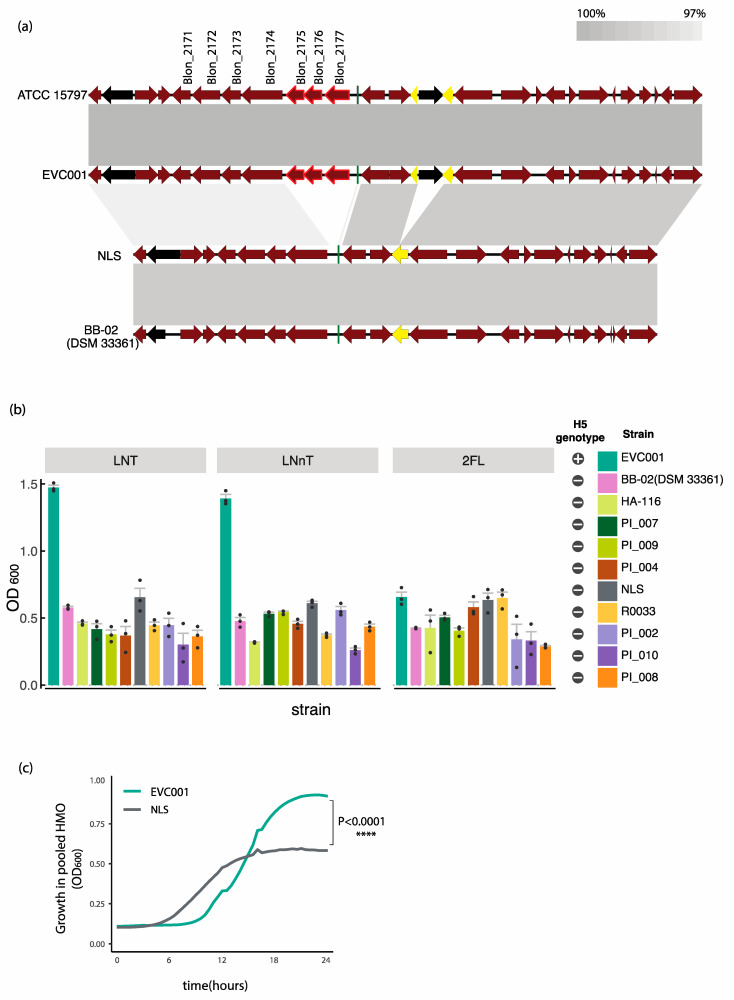
Alignment of a genomic region containing the H5 gene cluster and growth on purified HMOs from pooled human milk samples. (**a**) Genomic map of the H5 cluster and flanking of representative Group 1 (EVC001) and Group 2 (NLS and BB-02) strains compared to type strain. Genes belonging to the H5 cluster in type strain ATCC 15697 are annotated with the corresponding “Blon_” locus tag. Homologous segments are indicated by greyscale blocks. Genes absent in the NLS strain are highlighted in red. tRNAs are colored in green, truncated genes are colored in yellow and mobile genetic elements are colored in black. (**b**) Growth of *B infantis* strains isolated from infant probiotics on MRS medium containing lacto-*N*-tetraose (LNT), lacto-*N*-neotetraose (LNnT), or 2′-fucosyllactose (2′-FL) as the carbon source. Data represents the mean ± SD of three independent measurements. H5 genotype is indicated with a plus (+; H5 positive) or a minus sign (−; H5 negative). Colors representing the strains are correspond to the circular genome map in Figure 1. (**c**) Growth curves of *B. infantis* EVC001 and *B. infantis* NLS growing in RPMI medium containing purified HMOs from donor breast milk, **** *p* < 0.0001.

In *B. infantis,* genes related to HMO-utilization are organized in five clusters of genes (H1–H5) [14]. In order to determine the degree of conservation of these genes among strains, gene sequences were compared to the type strain *B. infantis* ATCC 15697 using tBLASTn (Figure 1b). Genetic differences were identified at the H1, H2 and H5 clusters. Specifically, the strain BB-02 (DSM 33361) was divergent from the type strain at the H1 cluster in the F1SBP genes Blon_2352 (80.15% identity) and Blon_2331 (90.4% identity). Furthermore, homologous sequences to the genes to Blon_0243 (a predicted transcriptional regulatory protein), Blon_0244 (a predicted histidine kinase HisK), and Blon_0245 (a predicted MFS1 transmembrane protein) of the H2 cluster were not identified in the genome of BB-02 (DSM 33361). The remaining Group 2 strains were divergent from the type strain at the H1 cluster genes Blon_2344, Blon_2347 and Blon_2351 (85.26% identity, 88.89% identity and 90.65% identity, respectively). The most consistent genomic divergence between strains in Group 1 and Group 2 occurred in the H5 cluster. Specifically, in the genes Blon_2175, Blon_2176 to Blon_2177 encoding an ABC-type transporter predicted to uptake lacto-*N*-biose (Figure 1b). Further examination, by aligning this genomic region in representative strains from Group 1 (ATCC 15697 and EVC001) and Group 2 (NLS and BB-02(DSM 33361)) (Figure 2a), revealed that the genes Blon_2175, Blon_2176 and Blon_2177 are in fact absent from the Group 2 strains (Figure 2a). This same region in the EVC001 and ATCC 15697 strains presented a high degree of synteny and conserved gene organization. Mobile genetic elements including a phage shock protein (Blon_2168) and transposon integrase (Blon_2181) were identified in the flanking regions to the ABC transporter in Group 1 strains. (Figure 2a). The genomes of NLS and BB-02 (DSM 33361) are predicted to encode genes homologous to Blon_2168 but not to Blon_2181 and possess an intact anti-terminator, which is truncated by the integration of Blon_2181 in Group 1 strains (Figure 2a). From these analyses, it can be concluded that the genomes of the analyzed *B. infantis* isolates display three distinct genomic variants with respect to HMO utilization genes. Group 1 strains are replete and possess the full gene repertoire of HMO-related genes (H5-positive genomic variant). Group 2 strains are all deplete of the ABC-type transporter of the H5 cluster (H5-negative genomic variant). The third genomic variant is displayed in the strain of BB-02 (DSM 33361), which in addition to being H5 negative is also deplete at the H2 cluster.

### 3.4. Divergence in the H5 Gene Cluster Is Associated with Poor Growth on HMOs

Probiotic strains were assessed for growth lacto-*N*-tetraose (LNT), lacto-*N*-neotetraose (LNnT), or 2′-fucosyllactose (2′-FL) as the sole carbohydrate source. These HMOs represent the major structural classes of neutral non-fucosylated (LNT and LNnT) and fucosylated (2′-FL) HMOs in human milk. At the 16 h time point, the mean OD_600_ of the H5-positive strain EVC00 on LNT (1.47 ± 0.03) and LNnT (1.39 ± 0.05) was more than twice as high as the mean OD_600_ reached by any of the H5-negative strains. Contrary to this trend, no major differences were observed between H5-positive and H5-negative strains growing in 2′-FL, with all mean OD_600_ values below 0.65 (Figure 2b).

To determine growth on a broader array of HMO structures present in human milk, representative strains from Group 1 (EVC001) and Group 2 (NLS) were grown on purified HMOs isolated from pooled human breast milk. Strains were selected on the basis that they represent the two identified H5-positive and H5-negative genomic variants of the H5 cluster and were available as single-strain probiotic products, facilitating subsequent feeding trials. As shown in Figure 2c, the Group 1 strain EVC001, which is H5 positive, reached significantly higher cell densities (*p* < 0.0001; two-way repeated-measures ANOVA) than the H5-negative NLS strain (Figure 2c).

**Figure 3:**
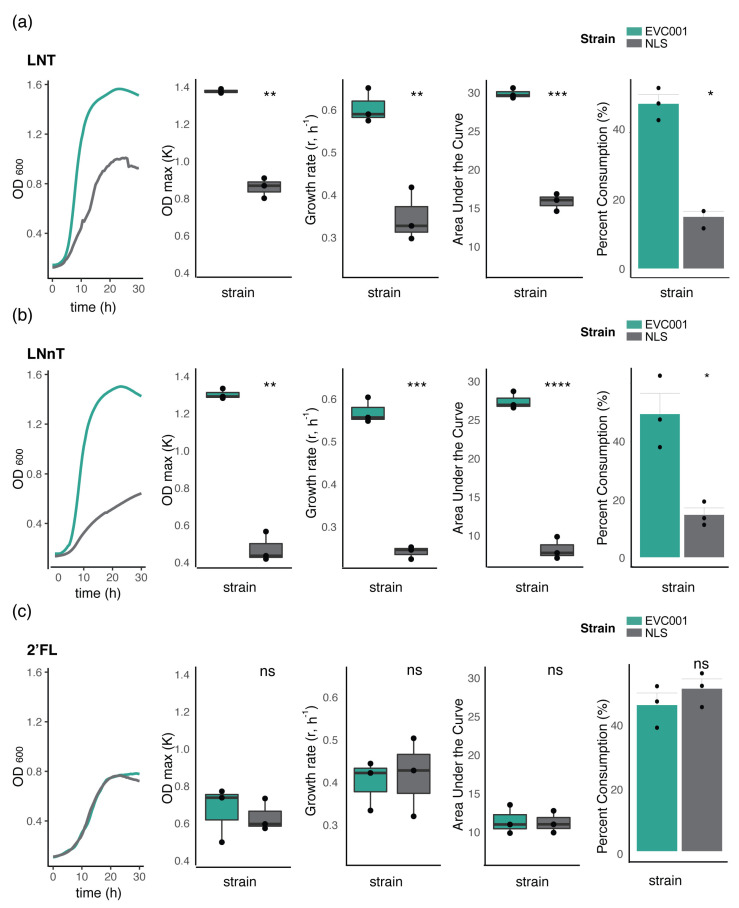
Growth on HMOs of select *B. infantis* strains. Growth kinetics and HMO consumption of *B. infantis* EVC001 (H5 positive, Group 1) and *B. infantis* NLS (H5 negative, Group 2) in mMRS medium containing (**a**) lacto-*N*-tetraose (LNT), (**b**) lacto-*N*-neotetraose (LNnT), or (**c**) 2′-fucosyllactose (2′-FL) as the carbon source. Growth indicators were compared, with statistical significance determined by Student’s *t*-test indicated with asterisks; ns: not significant, * *p* < 0.05, ** *p* < 0.01, *** *p* < 0.001 and **** *p* < 0.0001.

Because *B. infantis* has shown preferential consumption of smaller HMOs (degree of polymerization [DP] < 7), and to further characterize phenotypic implications to the variations of the H5 cluster, growth kinetics of select strains (EVC001 and NLS) grown on individual HMO standards was determined. This analysis revealed HMO-specific variation in the growth capacity and overall growth kinetics between strains. On LNT and LNnT, the H5-positive strain EVC001 attained significantly higher cell densities, at a faster growth rate and producing a significantly larger area under the curve (Figure 3a,b). The observed differences in growth were verified by glycoprofiling of the spent media, measuring the percent of HMOs consumed by individual bacterial strains. EVC001 consumed 48.4 ± 4.7% and 49.1 ± 12.4% of LNT and LNnT, respectively. This is significantly more (*p* < 0.05; Wilcoxon rank-sum test) than the percent consumption of NLS (15.2 ± 2.9% of LNT and 14.6 ± 4.11% LNnT). Notably, low amounts of free N-acetylglucosamine (0.34 ± 0.11 mg/mL) were detected in the spent media of NLS grown on LNT, which could be indicative of partial hydrolysis from lysed cells. Contrary to the findings with LNT and LNnT, growth kinetics in 2′-FL were indistinguishable between strains and the percent consumption of 2′-FL was comparable (44.7 ± 6.43% and 49.7 ± 5.23% for EVC001 and NLS, respectively) (Figure 3c). Notably, growth in this substrate promoted a moderate growth (max OD_600_ (k) = 0.68 ± 0.02) at a slower growth rate ((r) = 0.34 h^−1^ ± 0.12) for EVC001 compared to the vigorous growth on LNT (max OD_600_ = 1.3 ± 0.13; growth rate (r) 0.48 h^−1^ ± 0.17), and LNnT (max OD_600_ ± 0.09; growth rate (r) 0.42 h^−1^ ± 0.06). Overall, these results indicate that the Group 2 NLS strain, which lacks the ABC-type transporter genes of the H5 cluster, is significantly impaired, though not completely abrogated for growth on LNT and LNnT. Furthermore, results showed that growth differences are HMO specific, as the growth kinetics of both strains were congruent in 2′-FL.

### 3.5. Competitive Assays

**Figure 4:**
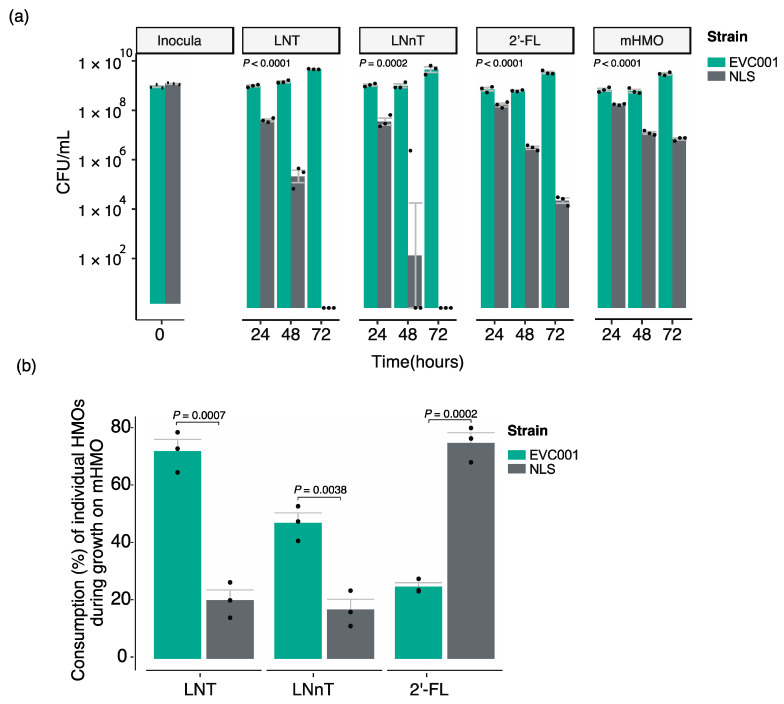
(**a**) *In vitro* competition experiments between the *B. infantis* strains EVC001 and NLS in the HMO standards lacto-*N*-tetraose (LNT), lacto-*N*-neotetraose (LNnT), 2′-fucosyllactose (2′-FL), or an equal combination of all three (mixed HMO (mHMO)). (**b**) Percent consumption of individual HMO standards by *B. infantis* EVC001 and *B. infantis* NLS during growth on mHMO. Statistical significance of strains in competition was determined by two-way repeated-measures ANOVA with Holm–Sidak multiple comparison adjustment. Significant differences in percent consumption were determined by Student’s *t*-test.

A competitive co-inoculation model was used to investigate the relative contribution of the H5 ABC-transporter to the ability of strains to grow under competitive conditions. As shown in Figure 4a, despite the starting inoculum containing comparable numbers of each strain (mean Log_10_ CFU/mL for EVC001 = 8.76 ± 0.07, and for NLS = 8.90 ± 0.03), the H5-positive EVC001 strain attained higher growth after the first passage in all three HMOs. The differences were, however, HMO-dependent. In the media containing LNT and LNnT, after 72 h in competition, the H5-negative NLS strain was completely outgrown by the H5-positive EVC001 (Figure 4a). In media containing 2′-FL, the strains maintained comparable growth for the first 24 h (mean Log_10_ CFU/mL for EVC001 = 8.82 ± 0.088, and for NLS = 8.12 ± 0.098). However, the trend favored EVC001 by the second passage, with the EVC001 reaching > 10-fold greater over the NLS strain (mean Log_10_ CFU/mL for EVC001 = 8.83± 0.036, and for NLS = 6.99 ± 0.908). By the third passage (72 h in competition), the mean Log_10_ difference between the strains had increased to 5 (mean Log_10_ CFU/mL for EVC001 = 9.53± 0.07, and for NLS = 4.34 ± 0.18). Intriguingly, the growth difference in 2′-FL observed in competition (Figure 4a) was incongruent with single-strain growth measurements (Figure 3c), which suggested strains were equally equipped to utilize this HMO. Finally, contrary to the results with the individual HMOs, when all three were combined (mHMO), the differences in growth between the strains were less prominent. This can be explained by the differences in hierarchical utilization of resources of the strains, in which EVC001 shows preference for LNT and LNnT, and NLS shows preference for 2′-FL (Figure 4b).

**Figure 5:**
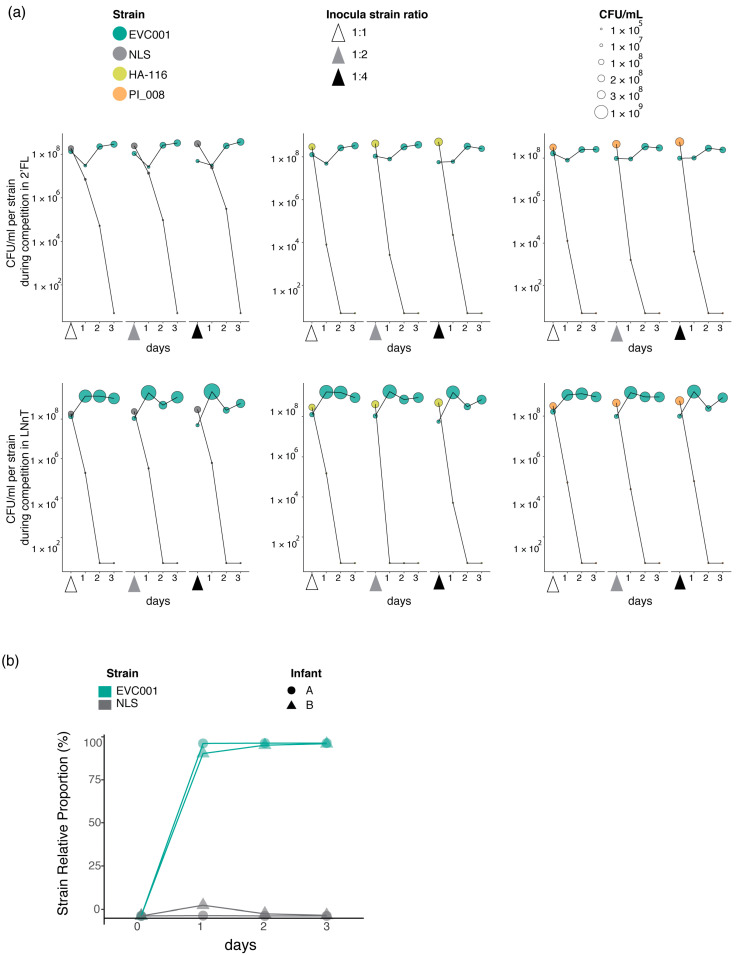
*In vitro* competition experiments and strain fitness in the breastfed infant gut. (**a**) *In vitro* growth experiments with varying rations of H5-negative to H5-positive strains in the inocula. Data represents the mean CFU/mL from three replicates. (**b**) Relative fitness (determined as proportion) of *B. infantis* NLS and *B. infantis* EVC001 in the fecal samples of two exclusively breastfed infants (A and B) before (day zero) and for three days after feeding the probiotic strains. For data analysis, if threshold cycle values were below detection limit, the bacterial abundance was recorded as the lowest dilution in the standard curve.

Co-inoculation competition experiments with varying strain ratios in the inoculum, were performed to determine whether the starting inoculum had an impact on the observed ecological fitness of strains, as well as to determine if the fitness disadvantage observed for NLS was true for other H5-negative strains. For this experiment, we choose LNnT and 2′-FL, as these were the HMOs for which the growth differences between EVC001 and NLS had been most prominent and most comparable, respectively. Mixtures were prepared at 1:1, 1:2, and 1:4 ratios, favoring the H5-negative strain. Results from this experiment show that the initial inoculum did not alter the observed competitive fitness of strains. Specifically, the H5-positive EVC001 outcompeted all three H5-negative strains (NLS, HA-116 and PI_008) by the third day of competition despite being at a numerical disadvantage in the inocula. These competitive dynamics do not appear to be strain or HMO-specific. As shown in Figure 5a, all H5-negative strains were detected 24 h after the initial inoculation but were eventually outcompeted by the second or third day of competition. The only exception to this trend was for the strain HA-116, which became undetected after 24 h in competition when inoculated 1:2 with EVC001. Furthermore, following the trend observed in the previous competition experiments using larger volumes, the strain NLS appeared to be more adept at competing for growth with EVC001 on 2′-FL. This is evidenced by the close cell counts (CFU/mL) between EVC001 and NLS by 24 h in this media. Specifically, 24 h after the strains were inoculated at a 1:1 ratio, the cell numbers were on average 3.0 × 10^7^ CFU/mL for EVC001 and 8.24 × 10^6^ CFU/mL for NLS, Similarly, after the inoculum at the 1:2 ratio, cell numbers were 2.23 × 10^7^ CFU/mL for EVC001 and 2.87 × 10^7^ CFU/mL for NLS. Finally, for the inoculum at the 1:4 ratio, at the 24 h time point, the detected cell numbers for the NLS strain were almost twice as much (3.76 × 10^7^ CFU/mL compared to 2.26 × 10^7^ CFU/mL) as EVC001. However, on the second day of the competition growth experiment in 2′-FL (48 h after initial inoculum), cells numbers for the NLS strain were considerably less, on average, when compared to EVC001, 2.26 × 10^8^ CFU/mL (NLS) vs. 5.18 × 10^4^ CFU/mL (EVC001) for the 1:1 inoculum; 2.60 × 10^8^ CFU/mL vs. 9.43 × 10^4^ CFU/mL for the 1:2 inoculum; and 2.43 × 10^8^ CFU/mL vs. 3.13 × 10^5^ CFU/mL for 1:4 inoculum. By day 3, the NLS became undetectable by qPCR in this media. Similar dynamics were not observed in LNnT, where the highest cell numbers for NLS after 24 h in competition were on average 6.19 × 10^5^ CFU/mL 24 h after the initial 1:4 inoculum compared to 2.48 × 10^9^ CFU/mL for EVC001. By the second day in competition for LNnT, the NLS strain was no longer detected in the media while EVC001 maintained populations of at least 10^7^ and 10^8^ on days 2 and 3 of competition, irrespective of the inoculum. The highest cell numbers, irrespective of inoculum, detected for the strain HA-116 after 24 h in competition with EVC001 were 3.39 × 10^4^ CFU/mL on 2′-FL and 4.98 × 10^5^ CFU/mL on LNnT. Similarly, irrespective of the inoculum, cell numbers of the strain PI_008 were detected at an average of 3.42 × 10^4^ CFU/mL on 2′-FL and 1.07 × 10^4^ on LNnT, compared to the strain ECV001 which formed populations of at least 3.47 × 10^7^ after 24 h in competition with the strains. Both HA-116 and PI_008 were undetectable by day 3 (Figure 5a). Collectively, these results provide further evidence that H5-negative strains have a growth impairment on LNT and LNnT, indicating absence of the H5 cluster ABC transporter is a deleterious mutation, which imposes a fitness detriment when in competition with H5-positive strains growing on HMOs.

To test whether the results observed *in vitro* had relevance *in vivo*, we performed a proof-of-concept trial by feeding EVC001 and NLS to two breastfed infants and calculated the relative proportion of the strains recovered from stool samples using a strain-specific qPCR over a 3-day period (Figure 5b). By day 3, the total counts of total *B. infantis* were 5 × 10^10^ and 2.1 × 10^9^ CFU per µg of stool DNA in infant A and B, respectively. This indicates that the *B. infantis* can abundantly colonize and thrive in conditions of the infant, which is in line with previous feeding trials of the strain *B. infantis* EVC001 [21]. As shown in Figure 5b, neither strain was detected in stools collected prior to feeding *B. infantis*. After feeding the strains and over the 3-day sampling period, the proportions of each strain in the stool samples were considerably different, with the H5-negative strain NLS making up a small proportion of the *B. infantis* population in comparison to the H5-positive strain EVC001 (Figure 5b). These results, although limited in sample size, when coupled with the *in vitro* data, suggest that genotypic differences of ABC-type transporter in the H5 cluster may represent a fitness cost on the ability of strains to compete for HMOs in the infant gut.

## 4. Discussion

The beneficial effects provided by *B. infantis* are attributed to its adaptation to colonizing the infant gut, which is mediated by the presence of specialized ABC transporters to capture and internalize HMOs [12]. Additionally, the overrepresentation of low-molecular-weight glycans that comprise breast milk HMOs and the corresponding preferential consumption of these glycans by *B. infantis* has long been speculated to be the result of a tripartite coevolutionary relationship between the mother’s milk, the infant, and *B. infantis* [13,47,48,49]. However, due to limited genetic tools for *B. infantis*, the traditional experimental validation of these hypotheses has been limited. In this study, we carried out a functional genomics approach to gain a mechanistic understanding of the metabolism of HMOs by *B. infantis* strains. We focused on the metabolism of neutral low-molecular-weight type-1 (LNT) and type-2 (LNnT) glycans as well as 2′-FL which are abundant in most human milk. We then conducted a small (*n* = 2) proof-of-concept trial aimed to test whether the findings *in vitro* could also be observed in the breastfed infant gut.

Analysis of the intra-species genomic diversity of twelve *B. infantis* strains isolated from commercial probiotic products revealed the genetic variability among strains, most of which consisted of hypothetical genes and mobile genetic elements. As previously reported [16], strain-level genetic variations were mainly located in genes predicted to be involved in the uptake of HMOs, particularly the Blon_2175-2177 ABC transport system located in the H5 cluster [16]. The Blon_2175-2177 ABC-transporter is predicted to bind core type-1 glycans (i.e., LNT) [50]. However, the relative gene expression of the solute binding protein Blon_2177 increases in the presence of LNT and LNnT [50,51] (Figure S1), which may suggest that is active on both type-1 and type-2 HMOs. Our results confirm and extend previous literature as we demonstrated that H5-negative strains, which lack the ABC transport system located in the H5 cluster, exhibited impaired growth when LNT, LNnT and pooled HMOs were provided as the sole carbon source (Figure 2, Figure 3, Figure 4 and Figure 5). Our results also indicated that the predicted inability of H5-negative strains to access these HMOs is associated with functional deficiencies *in vitro*. Furthermore, according to our preliminary *in vivo* data, respective to H5-positive strains, H5-negative strains are impaired in their ability to colonize the infant gut, which could have an impact on benefits imparted to the infant host. This highlights the importance of strain for the selection of probiotics for human milk-fed infants. However, appropriately controlled and powered trials are necessary to confirm this hypothesis. Future studies are also warranted to explore the effects of the oligosaccharide composition in human milk with respect to the colonization of *B. infantis* strains as well as compare the overall effects on the microbiome in infants fed H5-positive and H-5 negative strains.

The analyzed genomes of *B. infantis* encode numerous mobile genetic elements, suggesting the lateral acquisition of genetic material [14] (Figure 1a and Figure 2a). Horizontal gene transfer has long been recognized as an important force in the evolution of bacteria [52]. Changes to the genome by gene acquisition and deletion can confer selective advantages including the evolution of complex symbiotic traits [53] and host-specific adaptations [54,55]. Typically, horizontally acquired genetic regions are inserted near tRNA genes and are flanked by regions containing mobile elements such as phage related genes and transposases [52]. The presence of various phage-related sequences regions neighboring Blon_2175-2177 indicate that horizontal transfer events may have shaped this locus, as it has been detected in other regions of the genome of the type strain *B. infantis* ATCC 15697 [14]. However, the absence of intact prophage genes suggests that the integration did not occur recently. Moreover, the H5 cluster is the only HMO-related cluster conserved in members of three *B. longum* subspecies (*B. longum longum, B. longum infantis* and *B. longum suis*), providing further indication of an ancient HGT event, possibly predating the separation of the subspecies [16]. Thus, analysis of the H5 cluster in strains of these subspecies may provide insight into the events that have shaped the evolution of this genomic region. 

It remains unknown how a lineage of *B. infantis* strains lacking the Blon_2175-2177 ABC transporter emerged, and whether populations of this lineage are maintained naturally among infants. Our analysis was performed in strains isolated from probiotics, which given the high degree of genetic conservation are likely the same (individual) strain, distributed by various manufacturers, as has been reported for other probiotic bifidobacteria [34,56]. Further, although others have found similar genotypes in strains isolated from infant feces [16], it is possible for these strains to have originated from ingested probiotic products, as our own efforts to isolate *B. infantis* strains from control infants (not fed a *B. infantis* probiotic) from a previous clinical study in the US [21] were unsuccessful, but it agrees with results from other resource-rich countries where *B. infantis* is now absent [57]. This would suggest that the absence of the Blon_2175-2177 ABC transporter may be an artifact of the history of probiotic strains, which are known to be prone to phage infection and genome decay during production [58]). However, further experiments with a larger number of both probiotic-isolated and extant infant-derived strains will be required to evaluate this hypothesis. Finally, it has been argued that the amount and type of glycan fucosylation in breast milk and, in particular, the abundance of 2′-fucosyllation, directly impacts the colonization dynamics of bifidobacteria in infants from resource-rich geographical regions [58,59]. All strains characterized in this study encoded the complete set of genes predicted for 2′-FL transport (Blon_0341-0334 and Blon_2202-2204) [49]. Accordingly, the growth kinetics of the NLS strain during *in vitro* growth in 2′-FL remained very close to that of EVC001, indicating that both strains are similarly equipped to access this glycan (if present) in the breastfed infant gut; however, observed cell densities and growth rates were substantially lower during growth on 2′-FL than on LNT and LNnT (Figure 3), likely because fucose is not utilized by *Bifidobacterium* as a carbon source but rather metabolized into pyruvate and 1,2 propanediol [60]. This supports our previous observation that secretor status (i.e., FUT2 alleles) did not impact colonization by *B. infantis* EVC001 (Frese et al. 2017). Thus, 2′-FL may not be a critical substrate for *B. infantis* growth in the infant gut, relative neutral non-fucosylated to type-1 and type-2 HMOs, although 2’-FL might be relevant for interspecies syntrophic interactions within the microbiome [61]. Together, these findings indicate that LNT and LNnT are preferred substrates over 2′-FL for *B. infantis* strains whose genomes are replete with the full repertoire of HMO utilization genes.

While previous studies had predicted the role of the Blon_2175-2177 ABC transport system [50,51], results presented in this study specifically provide a mechanistic assessment of the role of this ABC transport system in the consumption of HMOs and in the colonization of the breastfed infant gut. Our results showed that the HMO consumption behavior of *B. infantis* is consistent with the functional ability inferred by the genomic analyses. The absence of this particular ABC transport system corresponded with impaired growth on LNT, LNnT and pooled HMOs from breast milk samples. LNT, LNnT and small (DP < 7) type-1 and type-2 HMOs are highly predominant in breast milk and are produced consistently throughout the lactation period [7]. Thus, the ability to access these glycans could be a major determinant in a strain’s competitive success in the breastfed infant gut.

Although our results strongly associate the Blon_2175-2177 ABC transport system with mediating the metabolism of LNT and LNnT, given that there is limited consumption of these HMOs by NLS (which lacks Blon_2175-2177), we cannot rule out the possibility that other less-efficient transporters located elsewhere in the genome participate in the uptake of these glycans. In fact, there are overlapping transcriptional signals and the expression of ABC permeases located outside the H5 cluster occur during growth on both LNT and LNnT, suggesting that the isomers are metabolized through overlapping pathways [50,51]. Additionally, although extracellular degradation of HMOs has not been described for *B. infantis*, the presence of trace amounts of intermediate monosaccharides in the spent media of the NLS strain could indicate partial degradation of LNT and LNnT, or the release of functional enzymes following cell lysis [19]. Further, the comparative genomics approach used in this study facilitated the explanation of differences previously observed in the HMO utilization phenotype of a number of *B. infantis* strains [50,51].

It is important to point out that while our results support a connection between a genomic variant and a fitness phenotype both *in vitro* and *in vivo,* our work is limited in that it cannot provide definitive conclusions to the substrate specificity of the H5 cluster ABC-type transporter. Significant progress has been made in the development of molecular tools for bifidobacteria [49]. Thus, future work undertaking loss of function gene knockout recovered through complementation will be required to derive definitive conclusions. Furthermore, although our *in vivo* results were consistent and in agreement with the *in vitro* findings, the competition experiments in breastfed infants are limited. Future, larger and well-powered studies are required to confirm the hypotheses generated in this study.

## 5. Conclusions

Given the importance of early life microbiome assembly for long-term health, and the role that *B. infantis* plays in providing key ecosystem services to the infant [62], there is a growing interest in the development of probiotic products containing *B. infantis*. Based on results presented here, we predict that strain-specific differences in the functional ability of *B. infantis* probiotics will also manifest in the colonization of the infant gut. These differences may have implications for benefits observed in human clinical studies linked to the high fitness of *B. infantis* EVC001, and the closely related ATCC 15697, in term and preterm infants [5,21,62,63,64,65,66]. In fact, the apparent lack of major functional differences between EVC001 and ATCC 15697 suggests that these strains share many phenotypic and health-promoting features related to the ability to metabolize HMOs and colonize the infant gut. Thus, our work provides important insights into the understanding of the symbiotic relationship between nursing infants and bifibacteria as well as key information regarding the ecology of *B. infantis*. This information may aid in the selection of strains and ingredients for probiotic and synbiotic formulations to support optimal modulation of the infant gut microbiome for the mitigation of risk for acute dysbiosis-related diseases such as NEC and, in the long term, risks associated with autoimmune disease development. In conclusion, our study highlights the importance of considering genotypic variations and functional properties of probiotics strains as potentially critical determinants of performance and fitness under the ecological conditions to which they are exposed.

## Figures and Tables

**Table 1:** General features of the bifidobacteria included in this study.

Group	Strain	Source Reference/Source	NCBI Accession Number	Size (Kbp)	Coverage (x)	GC (%)	CDSs	tRNAs
1	ATCC 15697	Sela (2008) sela [14]	NC_011593	2832	n/a	59.8	2547	84
1	JCM 1222	Fukuda (2011) Fukuda [20]	NC_017219	2828	n/a	59.8	2544	84
1	EVC001	probiotic product	NZ_LR655210	2832	147	59.8	2553	84
2	NLS	probiotic product	CP054528	2598	332	59.3	2226	59
2	BB-02 (DSM33361)	probiotic product	CP054527	2758	486	59.8	2437	60
2	PI_002	probiotic product	CP054526	2604	185	59.3	2227	59
2	HA-116	probiotic product	CP054525	2612	88	59.2	2232	59
2	PI_004	probiotic product	CP054524	2604	172	59.3	2226	59
2	R0033x	probiotic product “x”	CP054523	2614	130	59.2	2233	59
2	R0033y	probiotic product “y”	CP054596	2615	171	59.2	2234	59
2	PI_007	probiotic product	CP054522	2604	220	59.3	2228	59
2	PI_008	probiotic product	CP054521	2612	213	59.2	2230	59
2	PI_009	probiotic product	CP054520	2604	162	59.3	2230	59
2	PI_010	probiotic product	CP054519	2609	117	59.2	2231	59

NCBI: National Center for Biotechnology Informatio; %GC: percent of guanuce-cytosine; CDs: Coding Sequences; tRNAs: Transfer RNAs; n/a: not applicable.

## Supplementary Materials

The following are available online at https://www.mdpi.com/2072-6643/12/11/3247/s1, Table S1. Average Nucleotide Identity (ANI) of Bifidobacterium longum subps. infantis strians, Table S2. Putatively unique genes to Bifidobacterium longum subps. infantis BB-02 (DSM 33361), Figure S1. Relative expression of the solute binding protein Blon_2177 of *B. infantis* EVC001 grown on LNT and LNnT as the sole carbon source.
