# Supplementary material for: Comparative Genome Analysis of Bifidobacterium longum subsp. infantis Strains Reveals Variation in Human Milk Oligosaccharide Utilization Genes among Commercial Probiotics
Source: Nutrients. 2020 Oct 23;12(11):3247. doi: 10.3390/nu12113247 (PMC7690671; doi:10.3390/nu12113247)
Supplement: Supplementary file 1 [file nutrients-12-03247-s001.zip › supplementary materials/Figure S1.docx]

**Figure S1.** **Relative expression of the solute binding protein Blon_2177 of *B. infantis* EVC001 grown on LNT and LNnT as the sole carbon source.** Gene expression levels were determined by qPCR following methods in described in Garrido et. al (2010). Gene expression levels for of cells grown on lactose are shown as a reference

References:

Garrido, D.; Kim, J.H.; German, J.B.; Raybould, H.E.; Mills, D.A. Oligosaccharide binding proteins from *Bifidobacterium longum* subsp*. infantis* reveal a preference for host glycans. PLoS One 2011, 6, e17315, doi:10.1371/journal.pone.0017315.
